# Association between sleep apnea-specific novel hypoxic metrics and disturbances in glucose and lipid metabolism

**DOI:** 10.3389/fendo.2025.1691429

**Published:** 2026-01-22

**Authors:** Yujiao Zhang, Jiaqi Cai, Meirong Liu, Yan Wang, Haiyan Zhao, Jing Zhang

**Affiliations:** 1 Department of Respiratory and Critical Care Medicine, Tianjin Medical University General Hospital, Tianjin, China; 2 Department of General Internal Medicine, Tianjin University Tianjin Hospital, Tianjin, China; 3 Department of Sleep Medicine Center, the NO.983 Hospital of The People’s Liberation Army Joint Logistic Support Force, Tianjin, China

**Keywords:** disturbances in glucose and lipid metabolism, obstructive sleep apnea (OSA), percentage of sleep time with the duration of respiratory events causing desaturation (pRED_3p), sleep apnea-specific novel hypoxic metrics, sleep breathing impairment index (SBII)

## Abstract

**Objective:**

Obstructive sleep apnea (OSA) has been linked to disturbances in glucose and lipid metabolism. The independent association between sleep apnea–specific novel hypoxic metrics and metabolic dysregulation in OSA, however, remains unclear.

**Methods:**

Anthropometric and polysomnographic data were obtained from OSA patients treated. Novel hypoxic indices—percentage of sleep time with the duration of respiratory events causing desaturation (pRED_3p) and sleep breathing impairment index (SBII)—were derived from the SpO_2_ channel. Biochemical parameters were simultaneously assessed. Multiple linear regression models were applied to examine independent associations of pRED_3p, SBII, and apnea–hypopnea index (AHI) with glucose and lipid profiles. Logistic regression was further performed to estimate odds ratios (ORs) for abnormal glucose and lipid parameters across quartiles of pRED_3p, SBII, and AHI.

**Results:**

After controlling for confounding variables, pRED_3p demonstrated significant associations with fasting blood glucose (FBG) (β=0.122), fasting insulin (FIN) (β=0.410), HOMA-IR (β=0.325), total cholesterol (TC) (β=0.309), triglycerides (TG) (β=0.173), low-density lipoprotein cholesterol (LDL-C) (β=0.260), TC/HDL-C (β=0.182), TG/HDL-C (β=0.121), LDL-C/HDL-C (β=0.195), AI (β=0.182), LCI (β=0.663), visceral adiposity index (VAI) (β=0.115), and lipid accumulation product (LAP) (β=0.139). SBII was independently related to FBG (β=0.079), FIN (β=0.240), HOMA-IR (β=0.191), TC (β=0.179), TG (β=0.115), LDL-C (β=0.139), LDL-C/HDL-C (β=0.081), LCI (β=0.093), and LAP (β=0.098), all with *p<*0.05. In quartile-based comparisons, higher pRED_3p categories corresponded to progressively increased ORs for elevated FBG (2.372, 4.054, and 4.131), hyperinsulinemia (5.789, 22.644, and 26.188), high HOMA-IR (6.655, 36.637, and 43.807), hypercholesterolemia (2.751, 7.109, and 9.607), hypertriglyceridemia (1.976, 4.248, and 4.412), and elevated LDL-C (3.593, 8.050, and 12.048). Similarly, higher SBII quartiles were associated with increased ORs for elevated FBG (1.863, 3.819, and 3.874), hyperinsulinemia (5.002, 25.085, and 25.942), high HOMA-IR (5.879, 35.603, and 51.799), hypercholesterolemia (3.074, 7.297, and 8.867), hypertriglyceridemia (1.963, 4.101, and 4.757), and elevated LDL-C (3.627, 7.684, and 11.515). All linear trends were positive (*p<*0.001).

**Conclusion:**

pRED_3p and SBII were associated not only with established glucose and lipid metabolism parameters—including elevated FBG, FIN, HOMA-IR, hypercholesterolemia, hypertriglyceridemia, and elevated LDL-C—but also with composite lipid indices, exhibiting consistent linear trends. Greater OSA severity corresponded to more marked disturbances in glucose and lipid metabolism. These novel hypoxic metrics may serve as predictive indicators for metabolic dysregulation in OSA.

## 1 Introduction

Obstructive sleep apnea (OSA) is a prevalent sleep-related breathing disorder marked by recurrent upper airway obstruction, resulting in intermittent hypoxemia and repeated arousals. According to Benjafield et al. (1), reliable prevalence data for obstructive sleep apnea were available for 16 countries, from 17 studies, approximately 936 million adults aged 30–69 years worldwide exhibit mild to severe OSA, and about 425 million fall within the moderate to severe range, with the highest numbers in China, followed by the United States, Brazil, and India. Untreated OSA is associated with severe health consequences, including hypertension, cardiovascular disease (2), metabolic syndrome (MS) (3), and diabetes (4). Growing evidence has intensified interest in the association between OSA and MS, whose prevalence in OSA patients is estimated to be six to nine times higher than in the general population (5, 6). Dyslipidemia and impaired glucose metabolism, two defining features of MS, have been closely linked to adverse clinical outcomes in OSA (7), primarily attributed to intermittent nocturnal hypoxia and sleep fragmentation (8, 9). Clarifying the interplay between glucose–lipid metabolic disturbances and OSA holds substantial value for informing strategies in its clinical management and therapeutic intervention. The sleep breathing impairment index (SBII), introduced by Xiao Y and Cao WH’s team in 2022 (10), is calculated as the product of the duration of each obstructive event and the corresponding desaturation area, divided by total sleep time. This metric demonstrated a stronger correlation with elevated Framingham cardiovascular risk compared with the apnea-hypopnea index (AHI). In 2024, the same research group (11) proposed the pRED_3p, representing the proportion of cumulative apnea- and hypopnea-related sleep loss in the total sleep time, with the “3p” criterion designed to streamline area computation by excluding events with less than 3% desaturation. Their findings indicated that SBII was more predictive of mortality, whereas pRED_3p was more predictive of incidence in OSA-related CVD risk assessment. Nonetheless, the independent relationships between these OSA-specific hypoxic indices and glucose and lipid metabolic disturbances remain unverified. To address this gap, the present study analyzed anthropometric and polysomnographic data, derived novel hypoxic metrics including SBII and pRED_3p from the SpO_2_ channel, and obtained biochemical parameters. Multiple linear regression was employed to assess independent associations of SBII, pRED_3p, and AHI with glucose and lipid profiles, while logistic regression was applied to estimate ORs for glucose and lipid measures across quartiles of SBII, pRED_3p, and AHI.

## 2 Methods

### 2.1 Study design and population

**Figure 1:**
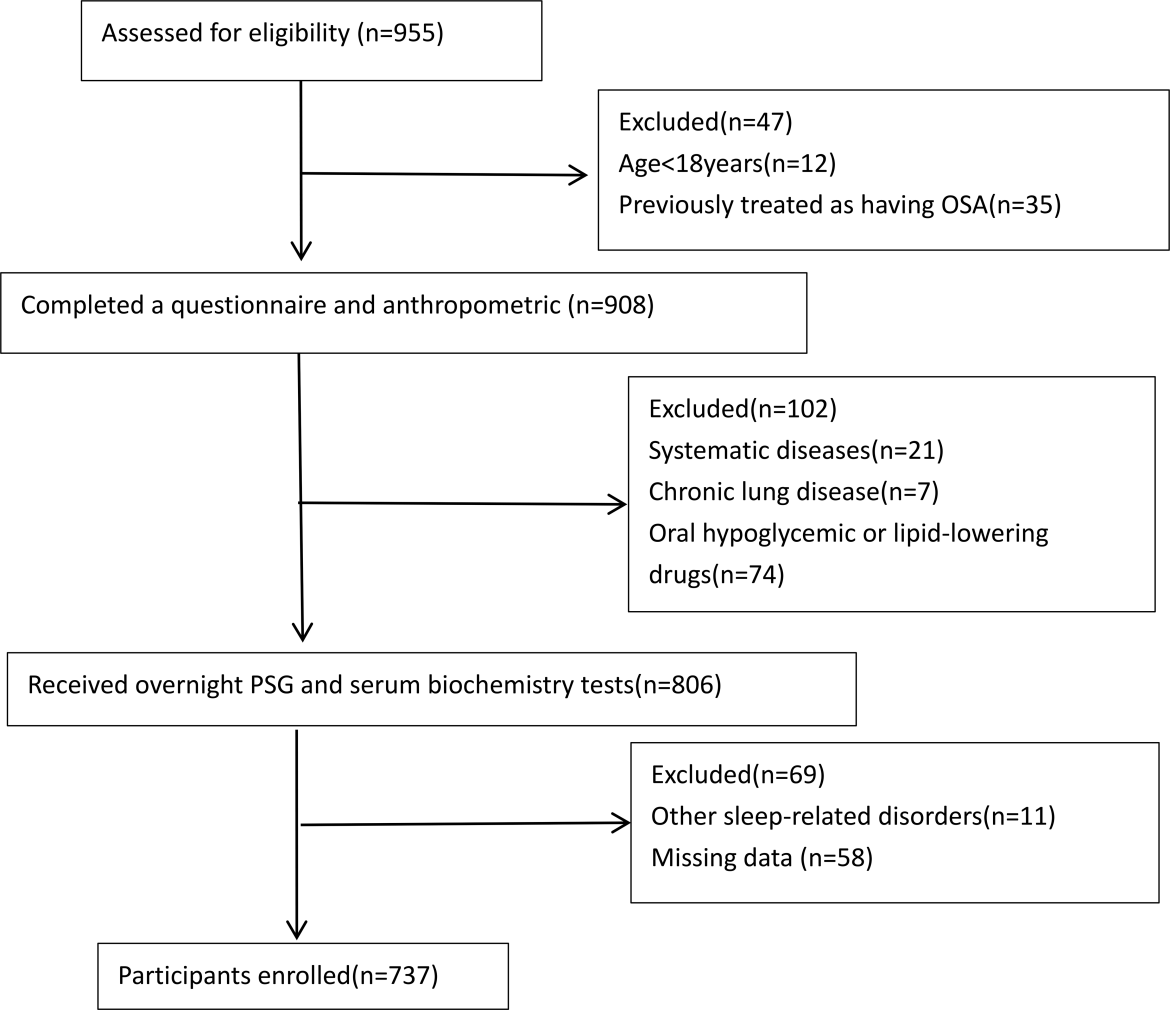
Participant screening flowchart.

Subjects suspected of OSA who underwent polysomnography at Tianjin Medical University General Hospital from January 2018 to June 2025 were retrospectively enrolled. Exclusion criteria included: (1) age <18 years; (2) ongoing OSA treatment; (3) severe systemic illness, such as hepatic or renal disease, renal failure, or malignancy; (4) respiratory disorders affecting oxygenation, including chronic obstructive pulmonary disease; (5) use of oral hypoglycemic or lipid-lowering drugs; (6) other diagnosed sleep disorders; and (7) incomplete data or inadequate polysomnography recording time. The recruitment process is illustrated in **Figure 1**. The study complied with the Declaration of Helsinki and received approval from the Institutional Review Board of Tianjin Medical University General Hospital (approval number: IRB2025-YX-249-01). Questionnaires and medical records were reviewed to obtain demographic and clinical parameters, including height, weight, neck circumference, waist circumference, neck-to-height ratio, BMI. Polysomnography data were analyzed to derive novel hypoxic indices, such as the SBII and pRED_3p, based on SpO_2_ measurements. Biochemical markers were also assessed.

### 2.2 Polysomnography and novel hypoxic metrics algorithm

Polysomnographic monitoring was conducted overnight using the Compumedics Grael system, with electrodes and sensors arranged in a standard diagnostic montage comprising six EEG leads (F4-M1, F3-M2, C4-M1, C3-M2, O2-M1, O1-M2), two electrooculographic leads (E1-M2, E2-M2), two submental EMG leads (chin1–chinZ, chin2–chinZ), bilateral anterior tibial EMG leads, ECG leads, oral and nasal thermocouples, nasal pressure transducers, uncalibrated thoracic and abdominal respiratory inductance plethysmography belts, a microphone-based snore sensor, Nonin finger pulse oximetry, body position sensors, and synchronized audio-video recording. Continuous overnight supervision ensured optimal signal integrity. The markers of respiratory events and the calculations of related sleep parameters were separately checked frame by frame by two skilled sleep technicians who performed the polysomnographic scoring to ensure reliability. At the same time, inter-rater reliability was assessed. Respiratory event scoring and parameter definitions adhered to the 2017 guidelines of the American Academy of Sleep Medicine (12).

Calculation of SBII and pRED_3p: We identified all manually calibrated apnea or hypopnea events, along with their respective desaturation search windows occurring within 100 seconds after the start of each event. The desaturation area associated with each respiratory event is calculated by integrating the cumulative desaturation areas within the designated search window (Figure 1 in Reference 11, top and middle). Artifacts related to oxygen desaturation events (default threshold: SpO_2_ < 20%) are detected and subsequently corrected by aligning them with proximate normal values (Figure 1 in Reference 11, bottom). The individual-specific SBII (%min^2^/h) is then calculated by dividing the sum of the products of all respiratory events and their corresponding desaturation areas by the total sleep duration. pRED_3p, quantifies the proportion of total sleep time occupied by the cumulative durations of sleep apneas and hypopneas. The suffix ‘3p’ indicates that this index exclusively considers events that coincide with oxygen desaturation levels of 3% or higher. This algorithm was developed by Xiao Y and Hui XJ’s group at Peking Union Medical College Hospital (11), who also released a Python script (version 3.9.6) on an open-source platform, enabling batch computation of SBII and pRED_3p.

### 2.3 Biochemical parameters

Blood samples were collected from the cubital vein on the morning following an overnight polysomnogram after an overnight fast to measure FBG, FIN, and lipid profiles, including TC, TG, HDL-C, and LDL-C. The lipid profiles and fasting glucose were measured by the Hitachi 7600 automatic biochemical analyzer, and fasting insulin was determined by chemiluminescence immunoassay. HOMA-IR was calculated as FIN (uIU/mL) × FBG (mmol/L)/22.5, with values ≥ 2.5 indicating insulin resistance (13, 14); hyperglycemia was defined as FBG ≥ 6.1 mmol/L, and hyperinsulinemia as FIN ≥ 12.2 uIU/mL (15, 16). Dyslipidemia was determined according to the US NCEPIII criteria (17): TC > 5.17 mmol/L, TG ≥ 1.7 mmol/L, HDL-C < 1.03 mmol/L, and LDL-C ≥ 3.37 mmol/L. Composite lipid indices included the TC/HDL-C, TG/HDL-C, and LDL/HDL ratios; non-HDL-C (TC − HDL-C); atherosclerosis index (AI) (non-HDL-C/HDL-C); lipoprotein combine index (LCI) (TC × TG × LDL-C/HDL-C); VAI (male: (WC/94) × (TG/1.03) ÷ (HDL-C/0.9) × (BMI/25); female: (WC/80) × (TG/0.81) ÷ (HDL-C/1.29) × (BMI/25)); and lipid accumulation product (LAP) (male: (WC − 65) × TG; female: (WC − 58) × TG).

### 2.3 Statistical analysis

Statistical analyses were conducted using SPSS 26. Normality was evaluated via the Kolmogorov-Smirnov test. Continuous variables with a normal distribution were expressed as mean ± standard deviation, whereas those without normal distribution were presented as medians with interquartile ranges. For normally distributed variables with homogeneity of variance, comparisons between two groups employed the T-test, and those among three or more groups employed the one-way ANOVA. Non-parametric methods were applied for variables lacking normality or equal variance. Linear trends between pRED_3p and SBII quartiles were assessed using Jonckheere-Terpstra for continuous variables and a Mantel-Haenszel linear association test for binary variables. Stepwise multiple linear regression was performed to determine independent associations of pRED_3p, SBII, and AHI with glucose metabolism indicators (FBG, FIN, and HOMA-IR) and lipid metabolism indicators, including both conventional (TC, TG, HDL-C, and LDL-C) and composite lipid profiles (TC/HDL-C, TG/HDL-C, LDL-C/HDL-C, AI, LCI, VAI, and LAP). Binary logistic regression identified risk factors for elevated FBG, hyperinsulinemia, insulin resistance, elevated TC, hypertriglyceridemia, reduced HDL-C, and elevated LDL-C. Linear trend evaluation incorporated quartile-specific values of pRED_3p, SBII, and AHI using binary logistic regression with polynomial contrast, and ORs with 95% CIs were computed. Collinearity diagnostics were conducted before analysis to exclude variables with multicollinearity (**Supplementary Tables S11**-**S19**). Model 1 was adjusted for sex (categorical), age, and BMI; Model 2 additionally included NHR and WHR; and Model 3 further accounted for STOP-Bang and MAP. pRED_3p, SBII, and AHI were analyzed both as continuous and categorical variables (quartiles); for the sake of simplification, linear regression results were reported only for continuous variables, and binary logistic regression results were presented exclusively for quartile-based categories. Statistical significance was set at a two-sided *p* < 0.05.

## 3 Results

### 3.1 Baseline characteristics and univariate analysis results by pRED_3p quartile

The analysis included 737 participants, comprising 488 males and 249 females. Stratification by pRED_3p quartile revealed significant variation in demographic parameters (sex, age, BMI, NHR, and WHR), sleep-related measures (STOP-Bang score, AHI), and MAP across quartiles (*p* for trend < 0.001, **Table 1**). In addition to HDL-C, other indicators of glucose and lipid metabolism, including FBG, FIN, HOMA-IR, TC, TG, LDL-C, TC/HDL-C, TG/HDL-C, LDL-C/HDL-C, AI, LCI, VAI, and LAP, also differed significantly, exhibiting a graded positive association with pRED_3p quartiles (*p* for trend < 0.01, **Table 1**). Moreover, the prevalence of elevated FBG, hyperinsulinemia, insulin resistance, high TC, high TG, and high LDL-C progressively increased with higher quartiles, ranging from 5.4% to 38.8%, 28.3% to 92.3%, 32.6% to 96.2%, 10.9% to 56.8%, 17.4% to 62.8%, and 6.0% to 45.4%, respectively (*p* for trend < 0.001, **Table 1**).

**Table 1:** Characteristics, sleep parameters, and biochemical indicators of patients divided by pRED_3p quartiles.

pRED_3p Groups	PRED_3p≤ 0.015	0.015 < pRED_3p ≤ 0.082	0.082 < pRED_3p ≤ 0.251	pRED_3p > 0.251	P-value for trend
numbers	184	188	182	183	--
Male, %	58.7	56.9	67	82.5	<0.001
Age,y	32 (23,45),	41 (29, 56)	44 (35,60)	45 (36,57)	<0.001
NHR	0.22 (0.21, 0.24)	0.23 (0.22, 0.25)	0.24 (0.23, 0.25)	0.25 (0.24, 0.26)	<0.001
WHR	0.53 (0.49, 0.58)	0.58 (0.54, 0.65)	0.61 (0.57, 0.67)	0.62 (0.59, 0.68)	<0.001
BMI, kg/m^2^	24.2 (22.0, 27.8)	26.4 (24.3, 31.4)	29.8 (26.7, 34.6)	30.4 (27.5, 34.7)	<0.001
STOPBang	2 (1, 3)	3 (2, 4)	4 (3, 5)	5 (4, 6)	<0.001
AHI	2.3 (1.0, 4.7)	11.7 (7.7, 16.9)	30.1 (21.6, 39.3)	64.1 (53.7, 77.2)	<0.001
MAP, mmHg	90 (83.67, 96.92)	95.33 (85.67, 103.33)	100.67 (90.92, 110.00)	112 (102.33, 123.33)	<0.001
FBG, mmol/L	4.83 (4.41, 5.22)	5.1 (4.8, 5.8)	5.6 (4.9, 6.6)	5.7 (4.9, 6.9)	<0.001
FIN, uIU/mL	7.8 (6.1, 13.7)	16.9 (11.8, 20.2)	21.6 (17.0, 28.9)	29 (20,36.5)	<0.001
HOMA-IR	1.77 (1.33, 2.96)	3.76 (2.57, 5.09)	5.26 (3.94, 7.57)	7.28 (4.87, 9.83)	<0.001
TC, mmol/L	3.97 (3.53, 4.57)	4.53 (4.05, 5.33)	5.16 (4.48, 5.85)	5.31 (4.82, 5.98)	<0.001
TG, mmol/L	1.13 (0.72, 1.55)	1.38 (0.97, 1.88)	1.80 (1.27, 2.75)	1.95 (1.46, 2.74)	<0.001
HDL-C, mmol/L	1.11 (0.96, 1.32)	1.08 (0.93, 1.26)	1.14 (0.98, 1.31)	1.10 (0.96, 1.24)	0.382
LDL-C, mmol/L	2.33 (1.82, 2.80)	2.72 (2.26, 3.24)	3.07 (2.60, 3.75)	3.29 (2.90, 3.84)	<0.001
Non-HDL-C, mmol/L	2.84 (2.42, 3.54)	3.44 (2.84, 4.08)	4.05 (3.32, 4.67)	4.16 (3.63, 4.91)	<0.001
TC/HDL-C	3.56 (2.91, 4.51)	4.18 (3.52, 5.03)	4.61 (3.88, 5.37)	4.77 (4.10, 5.70)	<0.001
TG/HDL-C	1.02 (0.62.1.52)	1.21 (0.84, 1.90)	1.62 (1.04, 2.51)	1.87 (1.27, 2.49)	<0.001
LDL-C/HDL-C	2.03 (1.56, 2.77)	2.58 (2.01, 3.12)	2.84 (2.14, 3.32)	3.05 (2.40, 3.75)	<0.001
AI	2.56 (1.91, 3.51)	3.18 (2.52, 4.03)	3.61 (2.88, 4.37)	3.77 (3.10, 4.70)	<0.001
LCI	9.07 (5.02, 16.53)	16.78 (9.77,26.83)	25.59 (16.47, 41.95)	32.38 (18.88, 52.10)	<0.001
VAI	1.52 (0.90, 2.63)	2.22 (1.28, 3.25)	2.52 (1.58, 4.00)	2.74 (1.90, 3.91)	<0.001
LAP	30.15 (15.84,50.81)	49.18 (31.44, 85.46)	78.60 (47.34, 135.62)	90.20 (61.07,150.48)	<0.001
Hyperglycemia,%	5.4	17.6	32.4	38.8	<0.001
Hyperinsulinemia,%	28.3	70.7	90.7	92.3	<0.001
HOMA-IR≥2.5,%	32.6	77.1	95.1	96.2	<0.001
Hyper TC, %	10.9	27.1	48.9	56.8	<0.001
Hyper TG, %	17.4	33.5	55.5	62.8	<0.001
HypoHDL-C, %	57.6	58.0	68.1	64.5	0.055
HyperLDL-C, %	6	19.7	35.7	45.4	<0.001
CVD, %	4.9	8.0	7.7	11.5	0.03
HBP, %	15.8	22.3	42.9	59.0	<0.001

BMI, body mass index; NHR, the ratio of neck circumference to height; WHR, the ratio of waist circumference to height; MAP, mean artierial pressure; FBG, fasting blood glucose; FIN, fasting insulin; HOMA-IR, insulin resistance index; TC, Total cholesterol; TG, Total triglycerides; HDL-C, High-density lipoprotein cholesterol; LDL-C, Low-density lipoprotein cholesterol; TC/HDL-C, the ratio of Total cholesterol to High-density lipoprotein cholesterol; TG/HDL-C, the ratio of Total triglycerides to High-density lipoprotein cholesterol; LDL-C/HDL-C,the ratio of Low-density lipoprotein cholesterol to High-density lipoprotein cholesterol; AI, atherogenic index; LCI, lipoprotein combine index; VAI, Visceral Adipose Index; LAP, Lipid Accumulation Product.

### 3.2 Relationship between pRED_3p and glucose and lipid metabolism

After controlling for confounding variables, pRED_3p exhibited significant associations with FBG (β=0.122), FIN (β=0.410), HOMA-IR (β=0.325), TC (β=0.309), TG (β=0.173), LDL-C (β=0.260), TC/HDL-C ratio (β=0.182), TG/HDL-C ratio (β=0.121), LDL-C/HDL-C ratio (β=0.195), AI (β=0.182), LCI (β=0.663), VAI (β=0.115), and LAP (β=0.139) (*p* for trend < 0.001, **Supplementary Tables S1**, **S2**). In quartile analysis, higher pRED_3p categories corresponded to progressively greater ORs for elevated FBG (2.372, 4.054, and 4.131; *p<*0.05), hyperinsulinemia (5.789, 22.644, and 26.188), high HOMA-IR (6.655, 36.637, and 43.807), hypercholesterolemia (2.751, 7.109, and 9.607), and hypertriglyceridemia (1.976, 4.248, and 4.412), while the ORs for elevated LDL-C were (3.593, 8.050, and 12.048, respectively). All observed linear trends were statistically significant (*p<*0.01) (**Supplementary Table S7**, Model 3).

### 3.3 Baseline characteristics and univariate analysis results by SBII quartile

By SBII quartile, significant differences were observed in demographic parameters (sex, age, BMI, NHR, and WHR), sleep-related indices (STOP-Bang score, AHI), and MAP (*p* for trend < 0.001, **Table 2**). In addition to HDL-C, continuous indicators of glucose and lipid metabolism, including FBG, FIN, HOMA-IR, TC, TG, LDL-C, TC/HDL-C, TG/HDL-C, LDL-C/HDL-C, AI, LCI, VAI, and LAP, also differed markedly across quartiles, exhibiting a graded positive association (*p* for trend < 0.01, **Table 2**). Moreover, the proportions of individuals with elevated FBG, hyperinsulinemia, insulin resistance, high TC, high TG, and high LDL-C increased progressively with higher SBII quartiles, ranging from 6.0% to 39.1%, 28.8% to 92.4%, 33.2% to 96.7%, 10.3% to 54.9%, 17.4% to 64.1%, and 6.0% to 45.1%, respectively (*p* for trend < 0.001, **Table 2**).

**Table 2:** Characteristics, sleep parameters, and biochemical indicators of patients by SBII quartiles.

SBII groups	SBII ≤ 1.615	1.615 < SBII ≤ 11.740	11.740 < SBII ≤ 47.854	SBII > 47.854	P-value for trend
numbers	184	185	184	184	--
Male, %	59.8	56.8	64.7	83.7	<0.001
Age, y	32 (22, 44)	42 (30,57)	44 (34,60’)	44 (36, 56)	<0.001
NHR	0.22(0.21,0.24)	0.23(0.22, 0.25)	0.24 (0.23, 0.25)	0.25 (0.24, 0.26)	<0.001
WHR	0.53(0.49,0.58)	0.58(0.54, 0.64)	0.61 (0.57, 0.67)	0.63 (0.59, 0.68)	<0.001
BMI, kg/m^2^	24.3(22.1,28.0)	26.4(24.2, 31.2)	29.4 (26.4, 34.6)	30.8 (27.7, 35.3)	<0.001
STOP Bang	2 (1, 3)	3 (2, 4)	4 (3, 5)	5 (4, 6)	<0.001
AHI	2.3 (1.0, 6.3)	11.6 (7.3, 16.7)	30.1 (21.1, 40.2)	64.1 (51.8, 77.2)	<0.001
MAP, mmHg	89.84 (83.42, 96.92)	95 (86.67, 103.17)	100.5 (91.17, 110)	112.34 (102.33, 123.33)	<0.001
FBG,mmol/L	4.8 (4.41, 5.22)	5.1 (4.8, 5.8)	5.6 (5.0, 6.7)	5.8 (4.9, 6.9)	<0.001
FIN, uIU/mL	7.8 (6.2, 14.0)	16.0(11.7, 20.1)	22 (17, 29)	29 (20,36)	<0.001
HOMA-IR	1.80(1.34,2.96)	3.75(2.51, 4.91)	5.43 (4.03, 7.64)	7.34 (4.86, 9.82)	<0.001
TC, mmol/L	3.96(3.53,4.57)	4.53(4.08, 5.35)	5.16 (4.46, 5.82)	5.28 (4.81, 5.98)	<0.001
TG, mmol/L	1.12(0.75,1.55)	1.4 (0.97, 1.88)	1.8 (1.28, 2.74)	1.97 (1.47, 2.73)	<0.001
HDL-C, mmol/L	1.11(0.96,1.32)	1.1 (0.94, 1.27)	1.12 (0.97, 1.29)	1.1 (0.95, 1.24)	0.613
LDL-C, mmol/L	2.36(1.82,2.84)	2.71(2.25, 3.27)	3.12 (2.58, 3.73)	3.28 (2.90, 3.84)	<0.001
Non-HDL-C, mmol/L	2.84(2.40,3.51)	3.47(2.86, 4.16)	4.05 (3.28, 4.64)	4.15 (3.58, 4.91)	<0.001
TC/HDL-C	3.58(2.91,4.51)	4.17(3.52, 5.02)	4.63 (3.89, 5.38)	4.76 (4.07, 5.70)	<0.001
TG/HDL-C	0.96(0.63,1.52)	1.18(0.83, 1.91)	1.66 (1.09, 2.45)	1.82 (1.32, 2.61)	<0.001
LDL-C/HDL-C	2.03(1.56,2.81)	2.58(1.96, 3.07)	2.84 (2.21, 3.38)	3.05 (2.41, 3.75)	<0.001
AI	2.58(1.91,3.51)	3.17(2.52, 4.02)	3.63 (2.90, 4.38)	3.76 (3.07, 4.70)	<0.001
LCI	9.07 (5.07,16.53)	16.71 (9.85,27.11)	25.49 (16.32,43.96)	32.26 (19.81, 52.10)	<0.001
VAI	1.52(0.92,2.63)	2.21(1.24, 3.18)	2.6 (1.67, 3.97)	2.69 (1.90, 3.88)	0.002
LAP	30.29 (16.45, 49.03)	49.44 (30.90, 85.24)	77.87 (46.50, 130.20)	91.73 (61.09, 153.40)	<0.001
Hyperglycemia,%	6	15.7	33.2	39.1	<0.001
Hyperinsulinemia, %	28.8	68.6	91.8	92.4	<0.001
HOMA-IR≥2.5,%	33.2	75.7	95.1	96.7	<0.001
Hyper TC, %	10.3	28.6	49.5	54.9	<0.001
Hyper TG, %	17.4	33	54.3	64.1	<0.001
Hypo HDL-C, %	57.1	60.5	65.8	64.7	0.080
Hyper LDL-C, %	6	20	35.3	45.1	<0.001
CVD, %	5.4	8.6	6.5	11.4	0.078
HBP, %	15.2	22.7	43.5	58.2	<0.001

BMI, body mass index; NHR, the ratio of neck circumference to height; WHR, the ratio of waist circumference to height; MAP, mean artierial pressure; FBG, fasting blood glucose; FIN, fasting insulin; HOMA-IR, insulin resistance index; TC, Total cholesterol; TG, Total triglycerides; HDL-C, High-density lipoprotein cholesterol; LDL-C, Low-density lipoprotein cholesterol; TC/HDL-C, the ratio of Total cholesterol to High-density lipoprotein cholesterol; TG/HDL-C, the ratio of Total triglycerides to High-density lipoprotein cholesterol; LDL-C/HDL-C, the ratio of Low-density lipoprotein cholesterol to High-density lipoprotein cholesterol; AI, atherogenic index; LCI, lipoprotein combine index; VAI, Visceral Adipose Index; LAP, Lipid Accumulation Product.

### 3.4 Relationship between SBII and glucose and lipid metabolism

After controlling for potential confounders, SBII demonstrated independent associations with FBG (β = 0.079), FIN (β = 0.240), HOMA-IR (β = 0.191), TC (β = 0.179), TG (β = 0.115), LDL-C (β = 0.139), LDL-C/HDL-C (β = 0.081), LCI (β = 0.093), and LAP (β = 0.098) (all *p* < 0.05; **Supplementary Tables S3**, **S4**, Model 3). Furthermore, relative to the lowest quartile, higher SBII quartiles were associated with elevated ORs for high FBG (1.863, 3.819, and 3.874), hyperinsulinemia (5.002, 25.085, and 25.942), high HOMA-IR (5.879, 35.603, and 51.799), hypercholesterolemia (3.074, 7.297, and 8.867), hypertriglyceridemia (1.963, 4.101, and 4.757), and elevated LDL-C (3.627, 7.684, and 11.515). All linear trends remained statistically significant at *p* < 0.01 (**Supplementary Table S8**, Model 3).

### 3.5 Relationship between AHI and glucose and lipid metabolism

Stratification by AHI quartiles revealed significant variations in demographic variables (sex, age, BMI, NHR, and WHR), sleep parameters (STOP-Bang score), MAP, FBG, FIN, HOMA-IR, TC, TG, LDL-C, TC/HDL-C, TG/HDL-C, LDL-C/HDL-C, AI, LCI, VAI, and LAP, demonstrating a graded positive association (*p* for trend <0.01, **Supplementary Table S10**). The prevalence of elevated FBG, hyperinsulinemia, insulin resistance, high TC, high TG, and high LDL-C progressively increased with higher AHI quartiles (*p* for trend <0.001, **Supplementary Table S10**). Multivariable analysis adjusting for potential confounders confirmed independent associations of AHI with FBG, FIN, HOMA-IR, TC, TG, LDL-C, TC/HDL, TG/HDL, LDL/HDL, AI, LCI, VAI, and LAP (all *p<*0.05, **Supplementary Tables S5**, **S6**, Model 3). In binary logistic regression, Model 3 indicated that the ORs and 95% CIs for glucose and lipid metabolic abnormalities exhibited a consistent positive linear gradient across AHI quartiles (all *p<*0.001, **Supplementary Table S9**, Model 3).

## 4 Discussion

MS is a multifactorial condition with considerable heterogeneity. Evidence from experimental, translational, and clinical research consistently indicates an association between OSA and multiple components of MS. This association is biologically plausible, primarily due to intermittent hypoxia (IH)—a defining feature of OSA—characterized by heightened sympathetic activation with consequent hemodynamic alterations, increased hepatic glucose production, insulin resistance mediated by adipose tissue inflammation, pancreatic islet cell dysfunction, hyperlipidemia through adverse fasting lipid profile changes, and reduced clearance of TG-rich lipoproteins. Although various mechanistic pathways have been proposed, the coexistence of visceral obesity and other confounding factors (e.g., pharmacologic interventions) complicates the determination of OSA’s independent role in MS (18). Given that SpO_2_ can be readily obtained from both laboratory-based and home sleep studies, incorporating IH depth and duration into prognostic models for disturbances in glucose and lipid metabolism has attracted increasing interest. Among emerging metrics, pRED_3p and SBII quantify both the severity and temporal extent of IH, demonstrating superior predictive performance for CVD compared with AHI, ODI, and hypoxic burden (11). Since disturbances in glucose and lipid metabolism are established determinants of cardiovascular morbidity and mortality, substantial efforts have been devoted to identifying parameters related to metabolic homeostasis (19). While pRED_3p and SBII have been validated as strong predictors of CVD, their relationship with glucose and lipid dysregulation remains unclear. In the present study, after stringent participant selection, analysis of objective polysomnography data, and comprehensive multivariable adjustment, compared with AHI, these new indicators of hypoxia have a stronger independent association with abnormal glucose and lipid metabolism. (See the results of multiple linear regression). Incremental increases in pRED_3p and SBII corresponded to a linear elevation in the risk of metabolic disturbances, including hyperglycemia, hyperinsulinemia, insulin resistance, hypercholesterolemia, hypertriglyceridemia, and elevated LDL-C (See the results of binary logistic regression).

Earlier investigations primarily examined the association between dyslipidemia and OSA in the context of conventional lipid parameters (20–24). More recent evidence indicates that composite lipid metrics—such as TC/HDL-C, TG/HDL-C, LDL-C/HDL-C, AI, and LCI—offer superior predictive value for cardiovascular disease compared with traditional profiles (25–27) and have increasingly been incorporated into OSA-related research (28, 29). The VAI serves as a robust marker for identifying individuals at elevated risk of metabolic disorders linked to visceral obesity, including insulin resistance, dyslipidemia, and cardiovascular risk factors (30, 31). Similarly, the LAP, calculated from waist circumference and TG levels, reflects abdominal lipid deposition and demonstrates strong predictive utility in clinical settings (32, 33). In this study, the analysis was extended to evaluate the associations between novel hypoxic indices and composite lipid parameters. pRED_3p exhibited independent correlations with TC/HDL-C, TG/HDL-C, LDL-C/HDL-C, AI, LCI, VAI, and LAP, while SBII was independently associated with LDL-C/HDL-C, LCI, and LAP, all with *p* < 0.05(See the results of multiple linear regression). These associations provide a basis for refining cardiovascular risk assessment and optimizing hyperlipidemia management strategies in patients with OSA.

In glucose metabolism, IH contributes to insulin resistance via β-cell dysfunction (34–36) and inflammation within adipose tissue (37–39). Specifically, it induces a pro-inflammatory phenotype in visceral adipose tissue (VAT), drives macrophage polarization toward the M1 subtype, and markedly increases the production and secretion of pro-inflammatory adipokines, thereby disrupting the insulin signaling pathway (37). IH also elevates hepatic glucose output (34, 35), impairs systemic glucose homeostasis, and promotes the onset of non-alcoholic fatty liver disease. In lipid metabolism, IH elevates TG and cholesterol concentrations by enhancing hepatic expression of key regulators, including SREBP-1 and SCD-1 (40). Furthermore, OSA-related hypoxia activates the sympathetic nervous system, which stimulates α-1 receptors to increase very low-density lipoprotein (VLDL) synthesis and suppresses hepatic clearance of LDL-C (41). Evidence indicates (42, 43) that α-1 receptor blockade raises HDL-C levels and reduces serum TG concentrations, whereas β-adrenergic receptor inhibition produces the opposite outcome. Additionally, norepinephrine and cortisol modulate HDL-C biosynthesis by influencing hormone-sensitive lipoproteins. Consequently, heightened sympathetic activity in OSA is linked not only to cardiovascular disease and adverse outcomes but also to the development of dyslipidemia (44). Collectively, these mechanisms contribute to reduced serum HDL-C and elevated TG, TC, and LDL-C levels in OSA, thereby increasing cardiovascular risk indicators such as TG/HDL-C ratio, LDL-C/HDL-C ratio, non-HDL-C, AI, and LCI (29).

Evidence indicates that sleep disruption acts as a physiological stressor, elevating hormones such as adrenocortical hormones and cortisol, which influence glucose and lipid catabolism and alter their metabolic profiles (45). In healthy individuals, sleep fragmentation not only can induce metabolic dysfunction directly but also modulate glucose and lipid metabolism indirectly through heightened sympathetic nervous system activity (46). pRED_3p was significantly correlated with 13 metabolic indicators, covering glucose homeostasis (FBG, FIN, HOMA-IR), lipid profile (TC, TG, LDL-C), and multiple composite risk indices. In contrast, SBII was only associated with 9 indicators. When comparing the β values of the same indicators, the effect size of pRED_3p was significantly larger than that of SBII. For instance, the impact on FIN: pRED_3p (β=0.410) was nearly 1.7 times that of SBII (β=0.240). The impact on LCI: pRED_3p (β=0.663) was more than 7 times that of SBII (β=0.093). The effects on HOMA-IR and LDL-C were also approximately twice as large as those of SBII. Based on the above results, we believe that pRED_3p performs better than SBII in predicting abnormal glycolipid metabolism. It is plausible that certain OSA patients, despite lacking marked hypoxia, still develop metabolic disturbances attributable to sleep fragmentation. Unlike SBII—which predominantly derives from desaturation-based calculations—pRED_3p identifies shorter respiratory events producing relatively minor desaturation, thereby capturing disturbances overlooked by SBII. Moreover, pRED_3p provides a direct measure of respiratory disturbance severity across the entire sleep period and partially reflects the temporal distribution of events. An increased cumulative duration of respiratory events throughout the night indicates more frequent interruptions and arousals, with greater sleep fragmentation correlating with more substantial adverse effects on glucose and lipid metabolism.

The strengths of this study include stringent participant selection, excluding individuals using glucose- or lipid-lowering medications, and the use of overnight polysomnography rather than portable monitoring, with all subjects achieving sleep durations exceeding six hours. In contrast to prior research that examined only one or two composite indicators, this analysis incorporated seven, enabling a broader scope of evaluation. Moreover, a multidimensional analytical framework was applied, with adjustments for potential confounders to enhance the robustness of the results. Nonetheless, certain limitations should be acknowledged. As a retrospective observational design, selection bias (such as excluding subjects receiving lipid-lowering or antidiabetic therapy, which narrows the clinical representativeness of the cohort), incomplete data capture (such as missing data), and lack of temporal causality indicate that the possibility of residual confounding cannot be excluded. Although rigorous exclusion criteria were applied regarding medication use, incomplete control over dietary pattern and exercise remains a limitation. The high correlation between pRED_3p and SBII may limit their independent interpretability. In addition, the analysis primarily emphasized indicators derived from respiratory and oxygen saturation channels, potentially overlooking other dimensions of OSA pathophysiology, including arousal threshold, loop gain, and muscle compensation.

## 5 Conclusions

pRED_3p and SBII demonstrate associations not only with conventional markers of glucose and lipid metabolism—including elevated FBG, fasting hyperinsulinemia, high HOMA-IR, hypercholesterolemia, hypertriglyceridemia, and elevated LDL-C—but also with composite lipid indices, exhibiting consistent linear relationships. Greater OSA severity corresponds to more marked disturbances in glucose and lipid metabolism. These novel hypoxic metrics may serve as predictive indicators for metabolic disturbances in OSA, which can improve the risk stratification of patients or serve as a potential marker for treatment response. This is of great clinical utility and can enhance the level of personalized medicine. If integrated into third-generation or portable devices, these novel hypoxia-related indicators could reliably reflect glucose and lipid metabolism profiles in OSA patients. This innovation would substantially reduce reliance on invasive venous blood sampling, offering a patient-friendly approach for continuous metabolic monitoring and management. Moreover, it holds the potential to streamline healthcare resource utilization by minimizing diagnostic costs.

## Data Availability

The original contributions presented in the study are included in the article/**Supplementary Material**. Further inquiries can be directed to the corresponding author/s.
