# Supplementary material for: Association between sleep apnea-specific novel hypoxic metrics and disturbances in glucose and lipid metabolism
Source: Front Endocrinol (Lausanne). 2026 Jan 22;16:1691429. doi: 10.3389/fendo.2025.1691429 (PMC12872566; doi:10.3389/fendo.2025.1691429)
Supplement: Supplementary file 1 [file Table1.docx]

**Supplemental file**

**Table S1** Stepwise multiple linear regression **(pRED_3p)** for glucose metabolism index in model 1, 2 and 3

|  | Reference | FBG, mmol/L | FIN,$u\text{U}/\text{mL}$ | HOMA-IR |
| --- | --- | --- | --- | --- |
| Model 1 | | | | |
| Sex | Female | — | -0.086(1.148)^C^ | -0.072(0.409)^C^ |
| Age, y |  | 0.194(0.003)^a^ | — | — |
| BMI |  | 0.237(0.007)^a^ | 0.099(0.077)^b^ | 0.131(0.027)^b^ |
| pRED_3p |  | 0.150(0.261)^a^ | 0.406(2.896)^a^ | 0.328(1.033)^a^ |
| Model 2 | | | | |
| Sex | Female | — | -0.089(1.189)c | -0.076(0.424)c |
| Age, y |  | 0.167(0.003)a | — | — |
| NHR |  | — | — | — |
| WHR |  | — | — | — |
| BMI |  | 0.143(0.01)c | — | — |
| pRED_3p |  | 0.149(0.261)a | 0.406(2.895)a | 0.328(1.032)a |
| Model 3 |  |  |  |  |
| Sex | Female | — | -0.095(1.369)c | — |
| Age, y |  | 0.139(0.004)b | — | — |
| NHR |  | — | — | — |
| WHR |  |  |  | — |
| BMI |  | — | — | — |
| STOP-Bang |  | — | — | — |
| MAP |  | — | — | — |
| pRED_3p |  | 0.122(0.29)b | 0.410(3.227)a | 0.325(1.150)a |

**Notes:** Data are presented as β (SE[β]). Model 1 adjusted for sex,age,BMI, and plus NHR,WHR in model 2 and plus STOP-Bang,MAP in model 3. ^a^p<0.001, ^b^p<0.01, ^c^p<0.05.**Abbreviations:** BMI, body mass index; NHR,

the ratio of neck circumference to height; WHR, the ratio of waist circumference to height;MAP, mean artierial pressure; FBG, fasting blood glucose; FIN, fasting insulin; HOMA-IR, insulin resistance index.

**Table S2**Stepwise multiple linear regression **(pRED_3p)** for Lipid profiles in model 1, 2 and 3

| Variable | Reference | | TC, mmol/L | | TG, mmol/L | HDL-C, mmol/L | | LDL-C, mmol/L | | TC/HDL-C | TG/HDL-C | | LDL-C/HDL-C | AI | | LCI | | VAI | | LAP |
| --- | --- | --- | --- | --- | --- | --- | --- | --- | --- | --- | --- | --- | --- | --- | --- | --- | --- | --- | --- | --- |
| Model1 | |  | |  | | |  | |  | | |  | | |  | |  | |  | |
| Sex | Female | | — | | — | -0.114(0.022)b | | — | | — | — | | — | — | | — | | — | | — |
| Age, y |  | | 0.136(0.002)a | | — | — | | 0.078(0.002)a | | — | — | | — | — | | — | | — | | — |
| BMI |  | | 0.121(0.006)b | | 0.137(0.008)b | — | | 0.178(0.005)^c^ | | — | — | | 0.168(0.005)a | — | | — | | — | | 0.450(0.353)a |
| pRED_3p |  | | 0.324(0.208)a | | 0.215(0.289)a | — | | 0.286(0.175)^a^ | | 0.194(0.374)^a^ | 0.130(0.689)b | | 0.224(0.200)a | 0.194(0.374)a | | 0.165(17.12)a | | 0.126(1.063)a | | 0.180(13.3)a |
|  | |  | |  | | |  | |  | | |  | | |  | |  | |  | |
| Sex | Female | | — | | — | -0.086(0.023)c | | — | | — | — | | — | — | | — | | — | | — |
| Age, y |  | | 0.127(0.003)a | | — | — | | — | | — | — | | — | — | | — | | — | | — |
| NHR |  | | — | | 0.127(2.667)b | -0.132(0.516)b | | — | | — | — | | 0.108(1.853)c | — | | — | | — | | 0.132(121.813)b |
| WHR |  | | — | | — | — | | — | | — | — | | — | — | | — | | — | | 0.113(37.482)c |
| BMI |  | | — | | — | — | | 0.115(0.007)c | | — | — | | — | — | | — | | — | | 0.284(0.508)a |
| pRED_3p |  | | 0.322(0.209)a | | 0.211(0.228)a | — | | 0.284(0.175)a | | 0.192(0.374)a | 0.130(0.69)b | | 0.222(0.2)a | 0.192(0.374)a | | 0.165(17.144)a | | 0.126(1.063)b | | 0.177(13.113)a |
| Model 3 |  | |  | |  |  | |  | |  |  | |  |  | |  | |  | |  |
| Sex | Female | | — | | — | -0.120(0.026)^b^ | | — | | — | — | | — | — | | — | | -0.099(0.502)c | | — |
| Age, y |  | | 0.126(0.003)b | | — | — | | — | | — | — | | — | — | | — | | — | | — |
| NHR |  | | — | | 0.113(2.753)c | -0.150(0.530)b | | — | | — | — | | 0.123(1.902)c | — | | — | | — | | 0.111(125.07)c |
| WHR |  | | — | | — | — | | — | | — | — | | — | — | | — | | — | | 0.117(37.496)c |
| BMI |  | | — | | — | — | | — | | — | — | | — | — | | — | | — | | 0.244(0.533)a |
| STOP-Bang |  | | — | | — | — | | — | | — | — | | — | — | | — | | — | | — |
| MAP |  | | — | | — | -0.102(0.001)c | | — | | — | — | | 0.099(0.003)c | — | | — | | — | | — |
| pRED_3p |  | | 0.309(0.232)a | | 0.173(0.32)a | — | | 0.260(0.195)a | | 0.182(0.417)a | 0.121(0.769)b | | 0.195(0.221)a | 0.182(0.417)a | | 0.663(19.108)a | | 0.115(1.184)c | | 0.139(14.557)a |

**Notes:** Data are presented as β (SE[β]). Model 1 adjusted for sex,age,BMI, and plus NHR,WHR in model 2 and plus STOP-Bang,MAP in model 3. ^a^p<0.001, ^b^p<0.01, ^c^p<0.05.

**Abbreviations:** BMI, body mass index; NHR, the ratio of neck circumference to height; WHR, the ratio of waist circumference to height;MAP, mean artierial pressure; TC, Total cholesterol; TG, Total triglycerides; HDL-C, High-density lipoprotein cholesterol; LDL-C, Low-density lipoprotein cholesterol;TC/HDL-C,the ratio of Total cholesterol to High-density lipoprotein cholesterol;TG/HDL-C,the ratio of Total triglycerides to High-density lipoprotein cholesterol; LDL-C/HDL-C,the ratio of Low-density lipoprotein cholesterol to High-density lipoprotein cholesterol; AI, atherogenic index; LCI, lipoprotein combine index; VAI,Visceral Adipose Index;LAP,Lipid Accumulation Product.

**Table S3** Stepwise multiple linear regression **(SBII)** for glucose metabolism index in model 1, 2 and 3

|  | Reference | FBG, mmol/L | FIN,$u\text{U}/\text{mL}$ | HOMA-IR |
| --- | --- | --- | --- | --- |
| Model 1 | | | | |
| Sex | Female | — | — | — |
| Age, y |  | 0.219(0.003)a | 0.074(0.034)c | 0.091(0.012)c |
| BMI |  | 0.259(0.007)a | 0.169(0.078)a | 0.188(0.027)a |
| SBII |  | 0.109(0.000)b | 0.268(0.006)a | 0.218(0.002)a |
| Model 2 | | | | |
| Sex | Female | — | — | — |
| Age, y |  | 0.192(0.003)a | — | — |
| NHR |  | — | — | — |
| WHR |  | — | — | — |
| BMI |  | 0.167(0.01)b | — | — |
| SBII |  | 0.108(0.000)b | 0.267(0.006)a | 0.217(0.002)a |
| Model 3 |  |  |  |  |
| Sex | Female | — | — | — |
| Age, y |  | 0.153(0.004)a | — | — |
| NHR |  | — | — | — |
| WHR |  |  |  | — |
| BMI |  | 0.125(0.011)c | — | — |
| STOP-Bang |  | — | — | — |
| MAP |  | — | — | — |
| SBII |  | 0.079(0.001)c | 0.240(0.006)a | 0.191(0.002)a |

**Notes:** Data are presented as β (SE[β]). Model 1 adjusted for sex,age,BMI, and plus NHR,WHR in model 2 and plus STOP-Bang,MAP in model 3. ^a^p<0.001, ^b^p<0.01, ^c^p<0.05.**Abbreviations:** BMI, body mass index; NHR, the ratio of neck circumference to height; WHR, the ratio of waist circumference to height;MAP, mean artierial pressure; FBG, fasting blood glucose; FIN, fasting insulin; HOMA-IR, insulin resistance index

**Table S4**Stepwise multiple linear regression **(SBII)** for Lipid profiles in model 1, 2 and 3

| Variable | Reference | TC, mmol/L | TG, mmol/L | | HDL-C, mmol/L | LDL-C, mmol/L | TC/HDL-C | | TG/HDL-C | LDL-C/HDL-C | | AI | LCI | VAI | LAP |
| --- | --- | --- | --- | --- | --- | --- | --- | --- | --- | --- | --- | --- | --- | --- | --- |
| Model 1 | |  | |  | |  | |  | | |  | |  |  |  |
| Sex | Female | — | — | | -0.114(0.022)b | — | 0.076(0.147)c | | — | 0.089(0.079)c | | 0.076(0.147)c | — | — | — |
| Age, y |  | 0.194(0.002)a | — | | — | 0.131(0.002)a | 0.082(0.004)c | | — | 0.080(0.002)c | | 0.082(0.004)c | — | — | — |
| BMI |  | 0.177(0.006)a | 0.169(0.008)a | | — | 0.230(0.005)a | 0.116(0.01)b | | — | 0.215(0.005)a | | 0.116(0.01)b | — | — | 0.474(0.346)a |
| SBII |  | 0.213(0.000)a | 0.157(0.001)a | | — | 0.181(0.000)a | 0.109(0.001)b | | 0.079(0.001)c | 0.124(0.000)a | | 0.109(0.001)b | 0.088(0.033)^a^ | — | 0.138(0.026)a |
| Model 2 | |  | |  | |  | |  | | |  | |  |  |  |
| Sex | Female | — | — | | -0.086(0.023)c | — | — | | — | — | | — | — | — | — |
| Age, y |  | 0.186(0.003)a | — | | — | 0.111(0.002)b | — | | — | — | | — | — | — | — |
| NHR |  | — | 0.135(2.694)^c^ | | -1.310(0.516)c | — | — | | — | 0.118(1.877)c | | — | — | — | 0.139(122.44)a |
| WHR |  | — | — | | — | — | — | | — | — | | — | — | — | 0.104(37.686)c |
| BMI |  | 0.160(0.008)b | — | | — | 0.170(0.007)b | — | | — | 0.124(0.008)c | | — | — | — | 0.311(0.503)a |
| SBII |  | 0.212(0.000)a | 0.156(0.001)a | | — | 0.180(0.000)a | 0.108(0.001)b | | — | 0.123(0.000)a | | 0.108(0.0.001)^a^ | 0.088(0.033)b | — | 0.136(0.025)a |
| Model 3 |  |  |  | |  |  |  | |  |  | |  |  |  |  |
| Sex | Female | — | — | | -0.119(0.026)^b^ | — | — | | — | — | | — | — | — | — |
| Age, y |  | 0.160(0.003)a | — | | — | 0.091(0.002)c | — | | — | — | | — | — | — | — |
| NHR |  | — | 0.115(2.767)c | | -1.149(0.530)b | — | — | | — | 0.123(1.923)c | | — | — | — | 0.112(125.554)c |
| WHR |  | — | — | | — | — | — | | — | — | | — | — | — | 0.111(37.611)c |
| BMI |  | 0.128(0.009)c | — | | — | 0.139(0.007)c | — | | — | — | | — | — | — | 0.256(0.532)a |
| STOP-Bang |  | — | — | | — | — | — | | — | — | | — | — | — | 0.100(2.064)c |
| MAP |  | 0.088(0.003)c | 0.093(0.004)c | | -0.099(0.001)c | 0.122(0.002)b | — | | — | 0.144(0.003)a | | — | — | — | — |
| SBII |  | 0.179(0.000)a | 0.115(0.001)b | | — | 0.139(0.000)a | — | | — | 0.081(0.000)c | | — | 0.093(0.035)c | — | 0.098(0.027)b |

**Notes:** Data are presented as β (SE[β]). Model 1 adjusted for sex,age,BMI, and plus NHR,WHR in model 2 and plus STOP-Bang,MAP in model 3. ^a^p<0.001, ^b^p<0.01, ^c^p<0.05.

**Abbreviations:** BMI, body mass index; NHR, the ratio of neck circumference to height; WHR, the ratio of waist circumference to height;MAP, mean artierial pressure; TC, Total cholesterol; TG, Total triglycerides; HDL-C, High-density lipoprotein cholesterol; LDL-C, Low-density lipoprotein cholesterol;TC/HDL-C,the ratio of Total cholesterol to High-density lipoprotein cholesterol;TG/HDL-C,the ratio of Total triglycerides to High-density lipoprotein cholesterol; LDL-C/HDL-C,the ratio of Low-density lipoprotein cholesterol to High-density lipoprotein cholesterol; AI, atherogenic index; LCI, lipoprotein combine index; VAI,Visceral Adipose Index;LAP,Lipid Accumulation Product.

**Table S5** Stepwise multiple linear regression **(AHI)** for glucose metabolism index in model 1, 2 and 3

|  | Reference | FBG, mmol/L | FIN,$u\text{U}/\text{mL}$ | HOMA-IR |
| --- | --- | --- | --- | --- |
| Model 1 | | | | |
| Sex | Female | — | -0.078(1.153)c | — |
| Age, y |  | 0.005(0.003)a | — | — |
| BMI |  | 0.036(0.007)a | — | 0.071(0.029)c |
| AHI |  | 0.009(0.002)a | 0.223(0.022)a | 0.063(0.008)a |
| Model 2 | | | | |
| Sex | Female | — | -0.080(1.195)c | — |
| Age, y |  | 0.013(0.003)a | — | — |
| NHR |  | — | — | — |
| WHR |  | — | — | — |
| BMI |  | — | — | — |
| AHI |  | 0.009(0.002)a | 0.223(0.022)a | 0.063(0.008)a |
| Model 3 |  |  |  |  |
| Sex | Female | — | -0.095(1.379)c | — |
| Age, y |  | 0.011(0.004)b | — | — |
| NHR |  | — | — | — |
| WHR |  |  |  | — |
| BMI |  | — | — | — |
| STOP-Bang |  | — | — | — |
| MAP |  | — | — | — |
| AHI |  | 0.008(0.002)a | 0.220(0.024)a | 0.061(0.009)a |

**Notes:** Data are presented as β (SE[β]). Model 1 adjusted for sex,age,BMI, and plus NHR,WHR in model 2 and plus STOP-Bang,MAP in model 3. ^a^p<0.001, ^b^p<0.01, ^c^p<0.05.**Abbreviations:** BMI, body mass index; NHR, the ratio of neck circumference to height; WHR, the ratio of waist circumference to height;MAP, mean artierial pressure; FBG, fasting blood glucose; FIN, fasting insulin; HOMA-IR, insulin resistance index.

**Table S6**Stepwise multiple linear regression **(AHI)** for Lipid profiles in model 1, 2 and 3

| Variable | Reference | TC, mmol/L | TG, mmol/L | | HDL-C, mmol/L | LDL-C, mmol/L | TC/HDL-C | | TG/HDL-C | LDL-C/HDL-C | | AI | LCI | VAI | LAP |
| --- | --- | --- | --- | --- | --- | --- | --- | --- | --- | --- | --- | --- | --- | --- | --- |
| Model 1 | |  | |  | |  | |  | | |  | |  |  |  |
| Sex | Female | — | — | | -0.068(0.022)b | — | — | | — | — | | — | — | — | — |
| Age, y |  | 0.008(0.002)a | — | | — | 0.004(0.002)c | — | | — | — | | — | — | — | — |
| BMI |  | — | 0.021(0.008)b | | -0.003(0.002)c | 0.015(0.005)b | — | | — | 0.019(0.006)a | | — | — | — | 0.427(0.372)a |
| AHI |  | 0.016(0.002)a | 0.012(0.002)a | | — | 0.011(0.001)a | 0.014(0.003)a | | 0.014(0.005)b | 0.009(0.002)a | | 0.014(0.003)a | 0.160(0.129)a | 0.021(0.008)b | 0.189(0.100)a |
| Model 2 | |  | |  | |  | |  | | |  | |  |  |  |
| Sex | Female | — | — | | -0.053(0.023)c | — | — | | — | — | | — | — | — | — |
| Age, y |  | 0.008(0.003)b | — | | — | — | — | | — | — | | — | — | — | — |
| NHR |  | — | 0.125(2.675)^c^ | | -0.133(0.516)b | — | — | | — | 0.106(1.851)c | | — | — | — | 0.131(121.802)b |
| WHR |  | — | — | | — | — | — | | — | — | | — | — | — | 0.113(37.469)c |
| BMI |  | — | — | | — | — | — | | — | — | | — | — | — | 0.263(0.520)a |
| AHI |  | 0.016(0.002)a | 0.012(0.002)a | | — | 0.011(0.001)a | 0.014(0.003)a | | 0.014(0.005)b | 0.009(0.002)a | | 0.014(0.003)a | 0.160(0.130)a | 0.021(0.008)b | 0.186(0.099)a |
| Model 3 |  |  |  | |  |  |  | |  |  | |  |  |  |  |
| Sex | Female | — | — | | -0.070(0.026)b | — | — | | — | — | | — | — | -0.097(0.503)c | — |
| Age, y |  | 0.008(0.003)b | — | | — | — | — | | — | — | | — | — | — | — |
| NHR |  | — | 0.111(2.75)c | | -0.151(0.529)b | — | — | | — | 0.120(1.9)c | | — | — | — | 0.109(124.974)c |
| WHR |  | — | — | | — | — | — | | — | — | | — | — | — | 0.117(37.47)c |
| BMI |  | — | — | | — | — | — | | — | — | | — | — | — | 0.225(0.541)a |
| STOP-Bang |  | — | — | | — | — | — | | — | — | | — | — | — | — |
| MAP |  | — | — | | -0.002(0.001)c | — | — | | — | 0.006(0.003)c | | — | — | — | — |
| AHI |  | 0.016(0.002)a | 0.01(0.002)a | | — | 0.010(0.001)a | 0.013(0.003)a | | 0.013(0.006)c | 0.008(0.002)a | | 0.013(0.003)a | 0.154(0.143)a | 0.018(0.009)c | 0.148(0.108)a |

**Notes:** Data are presented as β (SE[β]). Model 1 adjusted for sex,age,BMI, and plus NHR,WHR in model 2 and plus STOP-Bang,MAP in model 3. ^a^p<0.001, ^b^p<0.01, ^c^p<0.05.

**Abbreviations:** BMI, body mass index; NHR, the ratio of neck circumference to height; WHR, the ratio of waist circumference to height;MAP, mean artierial pressure; TC, Total cholesterol; TG, Total triglycerides; HDL-C, High-density lipoprotein cholesterol; LDL-C, Low-density lipoprotein cholesterol;TC/HDL-C,the ratio of Total cholesterol to High-density lipoprotein cholesterol;TG/HDL-C,the ratio of Total triglycerides to High-density lipoprotein cholesterol; LDL-C/HDL-C,the ratio of Low-density lipoprotein cholesterol to High-density lipoprotein cholesterol; AI, atherogenic index; LCI, lipoprotein combine index; VAI,Visceral Adipose Index;LAP,Lipid Accumulation Product.

**Table S7** Adjusted odds ratios for abnormal glucose and lipid metabolism according to pRED_3p categories in models 1, 2 and 3

|  | Hyperglycemia | Hyperinsulinemia | HOMA-IR≥2.5 | Hyper-total cholesterolemia | Hyper- triglyceridemia | Hypo-HDL cholesterolemia | Hyper-LDL cholesterolemia |
| --- | --- | --- | --- | --- | --- | --- | --- |
| Adjusted OR(95% CI) in model 1 | | | | | | | |
| pRED_3p≤0.015 | 1 | 1 | 1 | 1 | 1 | 1 | 1 |
| 0.015＜pRED_3p≤0.082 | 2.52(1.179,5.338)c | 5.740(3.531,9.330)a | 6.606(4.016,10.864)a | 2.819(1.569,5.068)a | 2.080(1.246,3.470)b | 0.979(0.632,1.516) | 3.650(1.757,7.581)a |
| 0.082＜pRED_3p≤0.251 | 4.535(2.135,9.630)a | 22.468(11.506,43.873)a | 36.535(16.324,81.766)a | 7.315(3.988,13.416)a | 4.684(2.720,8.064)a | 0.626(0.383,1.023) | 8.398(3.995,17.652)a |
| pRED_3p＞0.251 | 5.481(2.533,11.863)a | 28.605(13.761,59.462)a | 48.330(19.595,119.207)a | 10.544(5.570,19.595)a | 6.114(3.444,10.853)a | 0.664(0.396,1.113) | 13.270(6.143,28.665)a |
| P-value for linear trend | P<0.001 | P<0.001 | P<0.001 | P<0.001 | P<0.001 | 0.132 | P<0.001 |
| Adjusted OR(95% CI) in model 2 | | | | | | | |
| pRED_3p≤0.015 | 1 | 1 | 1 | 1 | 1 | 1 | 1 |
| 0.015＜pRED_3p≤0.082 | 2.482(1.158,5.322)c | 5.748(3.536,9.346)a | 6.644(4.036,10.935)a | 2.773(1.541,4.991)a | 2.046(1.220,3.431)b | 0.995(0.615,1.483) | 3.638(1.751,7.558)a |
| 0.082＜pRED_3p≤0.251 | 4.545(2.138,9.661)a | 22.574(11.554,44.105)a | 36.909(16.475,82.689)a | 7.258(3.954,13.323)a | 4.719(2.730,8.158)a | 0.612(0.373,1.002) | 8.381(3.998,17.617)a |
| pRED_3p＞0.251 | 5.384(2.481,11.681)a | 28.89(13.868,60.208)a | 49.540(19.981,122.826)a | 10.123(5.338,19.197)a | 5.853(3.283,10.435)a | 0.619(0.367,1.043) | 13.292(6.142,28.766)a |
| P-value for linear trend | P<0.001 | P<0.001 | P<0.001 | P<0.001 | P<0.001 | 0.098 | P<0.001 |
| Adjusted OR(95% CI) in model 3 | | | | | | | |
| pRED_3p≤0.015 | 1 | 1 | 1 | 1 | 1 | 1 | 1 |
| 0.015＜pRED_3p≤0.082 | 2.372(1.104,5.097)c | 5.789(3.154,9.467)a | 6.655(4.022,11.011)a | 2.751(1.526,4.958)a | 1.976(1.176,3.319)b | 0.962(0.618,1.496) | 3.593(1.728,7.470)a |
| 0.082＜pRED_3p≤0.251 | 4.054(1.889,8.698)a | 22.644(11.439,44.824)a | 36.637(16.163,83.047)a | 7.109(3.843,13.151)a | 4.248(2.440,7.394)a | 0.618(0.374,1.021) | 8.050(3.807,17.022)a |
| pRED_3p＞0.251 | 4.131(1.823,9.358)a | 26.188(11.837,57.941)a | 43.807(16.671,115.112)a | 9.607(4.856,19.006)a | 4.412(2.374,8.196)a | 0.631(0.357,1.115) | 12.048(5.367,27.045)a |
| P-value for linear trend | 0.007 | P<0.001 | P<0.001 | P<0.001 | P<0.001 | P=0.146 | P<0.001 |

Notes: ORs were adjusted for sex age, BMI inmodel 1, and plus NHR,WHR in model 2 and plus STOP-Bang,MAP in model 3. ap<0.001, bp<0.01, cp<0.05.

P-values for linear trends were determined by examining the median pRED_3p value for each quartile;

**Table S8** Adjusted odds ratios for abnormal glucose and lipid metabolism according to SBII categories in models 1, 2 and 3

|  | Hyperglycemia | Hyperinsulinemia | HOMA-IR≥2.5 | Hyper-total cholesterolemia | Hyper- triglyceridemia | Hypo-HDL cholesterolemia | Hyper-LDL cholesterolemia |
| --- | --- | --- | --- | --- | --- | --- | --- |
| Adjusted OR(95% CI) in model 1 | | | | | | | |
| SBII≤1.615 | 1 | 1 | 1 | 1 | 1 | 1 | 1 |
| 1.615＜SBII≤11.740 | 1.998(0.946,4.222) | 5.059(3.130,8.176)a | 5.942(3.635,9.713)a | 3.156(1.747,5.701)a | 2.078(1.244,3.472)b | 0.918(0.592,1.425) | 3.695(1.781,7.666)a |
| 11.740＜SBII≤47.854 | 4.296(2.080,8.875)a | 24.987(12.606,49.530)a | 35.373(15.909,78.649)a | 7.602(4.141,13.956)a | 4.556(2.658,7.807)a | 0.709(0.437,1.151) | 8.023(3.840,16.762)a |
| SBII＞47.854 | 5.065(2.397,10.705)a | 27.232(13.121,56.517)a | 53.635(20.733,138.753)a | 9.935(5.231,18.867)a | 6.445(3.630,11.445)a | 0.699(0.418,1.169) | 12.744(5.920,27.434)a |
| P-value for linear trend | P<0.001 | P<0.001 | P<0.001 | P<0.001 | P<0.001 | P=0.418 | P<0.001 |
| Adjusted OR(95% CI) in model 2 | | | | | | | |
| SBII≤1.615 | 1 | 1 | 1 | 1 | 1 | 1 | 1 |
| 1.615＜SBII≤11.740 | 1.966(0.928,4.163) | 5.057(3.128,8.174)a | 5.958(3.643,9.745)a | 3.120(1.725,5.644)a | 2.053(1.223,3.448)b | 0.901(0.579,1.402) | 3.674(1.770,7.652)a |
| 11.740＜SBII≤47.854 | 4.310(2.083,8.917)a | 25.099(12.656,49.775)a | 35.717(16.049,79.491)a | 7.554(4.110,13.884)a | 4.597(2.672,7.909)a | 0.695(0.427,1.131) | 8.012(3.836,16.737)a |
| SBII＞47.854 | 4.968(2.345,10.525)a | 27.406(13.178,56.996)a | 54.712(21.058,142.148)a | 9.553(5.02,18.179)a | 6.176(3.463,11.013)a | 0.654(0.389,1.1) | 12.737(5.908,27.462)a |
| P-value for linear trend | P<0.001 | P<0.001 | P<0.001 | P<0.001 | P<0.001 | 0.322 | P<0.001 |
| Adjusted OR(95% CI) in model 3 | | | | | | | |
| SBII≤1.615 | 1 | 1 | 1 | 1 | 1 | 1 | 1 |
| 1.615＜SBII≤11.740 | 1.863(0.877,3.959) | 5.002(3.077,8.130)a | 5.879(3.576,9.663)a | 3.074(1.696,5.571)a | 1.963(1.166,3.305)b | 0.914(0.586,1.426) | 3.627(1.746,7.535)a |
| 11.740＜SBII≤47.854 | 3.819(1.824,7.997)a | 25.085(12.444,50.564)a | 35.603(15.737,80.549)a | 7.297(3.933,13.538)a | 4.101(2.361,7.121)a | 0.712(0.433,1.171) | 7.684(3.649,16.177)a |
| SBII＞47.854 | 3.874(1.747,8.589)a | 25.942(11.695,57.541)a | 51.799(18.735,143.215)a | 8.867(4.467,17.601)a | 4.757(2.557,8.849)a | 0.678(0.384,1.197) | 11.515(5.145,25.774)a |
| P-value for linear trend | P=0.002 | P<0.001 | P<0.001 | P<0.001 | P<0.001 | P=0.457 | P<0.001 |

Notes: ORs were adjusted for sex age, BMI inmodel 1, and plus NHR,WHR in model 2 and plus STOP-Bang,MAP in model 3. ap<0.001, bp<0.01, cp<0.05.

P-values for linear trends were determined by examining the median SBII value for each quartile;

**Table S9** Adjusted odds ratios for abnormal glucose and lipid metabolism according to AHI categories in models 1, 2 and 3

|  | Hyperglycemia | Hyperinsulinemia | HOMA-IR≥2.5 | Hyper-total cholesterolemia | Hyper- triglyceridemia | Hypo-HDL cholesterolemia | Hyper-LDL cholesterolemia |
| --- | --- | --- | --- | --- | --- | --- | --- |
| Adjusted OR(95% CI) in model 1 | | | | | | | |
| AHI≤6.8 | 1 | 1 | 1 | 1 | 1 | 1 | 1 |
| 6.8＜AHI≤20 | 3.049(1.386,6.709)b | 6.773(4.157,11.034)a | 9.9(5.914,16.575)a | 2.419(1.32,4.431)b | 2.725(1.636,4.539)a | 0.749(0.487,1.152) | 3.136(1.539,6.39)b |
| 20＜AHI≤46.15 | 6.739(3.140,14.460)a | 26.134(13.55,50.404)a | 50.562(22.068,115.848)a | 9.558(5.235,17.452)a | 4.990(2.934,8.448)a | 0.647(0.407,1.029) | 8.482(4.2,17.129)a |
| AHI＞46.15 | 6.225(2.828,13.7)a | 34.185(16.229,72.008)a | 43.728(18.751,101.975)a | 13.777(7.262,26.135)a | 7.373(4.188,12.981)a | 0.549(0.333,0.906) | 11.661(5.602,24.273)a |
| P-value for linear trend | P<0.001 | P<0.001 | P<0.001 | P<0.001 | P<0.001 | P=0.118 | P<0.001 |
| Adjusted OR(95% CI) in model 2 | | | | | | | |
| AHI≤6.8 | 1 | 1 | 1 | 1 | 1 | 1 | 1 |
| 6.8＜AHI≤20 | 3.067(1.391,6.763)b | 6.787(4.164,11.06)a | 9.998(5.958,16.747)a | 2.415(1.318,4.427)b | 2.773(1.657,4.641)a | 0.745(0.483,1.148) | 3.128(1.535,6.376)b |
| 20＜AHI≤46.15 | 6.722(3.13,14.437)a | 26.252(13.6,50.673)a | 51.112(22.268,117.322)a | 9.431(5.164,17.224)a | 4.970(2.912,8.492)a | 0.628(0.394,1) | 8.454(4.186,17.074)a |
| AHI＞46.15 | 6.110(2.773,13.463)a | 34.497(16.347,72.999)a | 44.661(19.076,104.557)a | 13.332(7.027,25.295)a | 7.144(4.046,12.617)a | 0.515(0.310,0.854) | 11.642(5.586,24.265)a |
| P-value for linear trend | P<0.001 | P<0.001 | P<0.001 | P<0.001 | P<0.001 | P=0.071 | P<0.001 |
| Adjusted OR(95% CI) in model 3 | | | | | | | |
| AHI≤6.8 | 1 | 1 | 1 | 1 | 1 | 1 | 1 |
| 6.8＜AHI≤20 | 2.948(1.334,6.515)b | 7.153(4.341,11.788)a | 10.604(6.241,18.016)a | 2.421(1.319,4.444)b | 2.702(1.611,4.531)a | 0.745(0.482,1.15) | 3.092(1.516,6.306)b |
| 20＜AHI≤46.15 | 6.027(2.78,13.068)a | 28.687(14.428,57.04)a | 55.606(23.496,131.598)a | 9.549(5.180,17.602)a | 4.497(2.610,7.748)a | 0.623(0.387,1.004) | 8.043(3.954,16.361)a |
| AHI＞46.15 | 4.898(2.152,11.149)a | 36.665(16.352,82.212)a | 46.239(18.479,115.703)a | 13.616(6.936,26.728)a | 5.753(3.155,10.488)a | 0.508(0.294,0.877) | 10.494(4.897,22.488)a |
| P-value for linear trend | P<0.001 | P<0.001 | P<0.001 | P<0.001 | P<0.001 | P=0.097 | P<0.001 |

Notes: ORs were adjusted for sex age, BMI inmodel 1, and plus NHR,WHR in model 2 and plus STOP-Bang,MAP in model 3. ap<0.001, bp<0.01, cp<0.05.

1. values for linear trends were determined by examining the median AHI value for each quartile;

表S10、Characteristics, sleep parameters, and biochemical indicators of patients divided by AHI quartiles

|  | AHI≤6.8 | 6.8＜AHI≤20 | 20＜AHI≤46.15 | AHI＞46.15 | P for trend |
| --- | --- | --- | --- | --- | --- |
| numbers | 186 | 184 | 183 | 184 |  |
| Male,% | 54.3 | 62 | 68.9 | 79.9 | <0.001 |
| Age,y | 33(24,45) | 39(29,56) | 46(35,60) | 44（36,56） | <0.001 |
| NHR | 0.22(0.21,0.24) | 0.23(0.22,0.24) | 0.24(0.23,0.25) | 0.25（0.23,0.26） | <0.001 |
| WC,cm | 92(84,103) | 98(90,106) | 103(96,112) | 108（102,120） | <0.001 |
| WHR | 0.55(0.50,0.61) | 0.57(0.53,0.63) | 0.61(0.56,0.66) | 0.63（0.59,0.69） | <0.001 |
| BMI, kg/m^2^ | 25.3(22.6,29.9) | 26.5(24.1,30.4) | 28.7(25.5,32.9) | 30.9（27.7,35.5） | <0.001 |
| STOPBang | 2(1,3) | 3(2,4) | 4(3,5) | 5（3,6） | <0.001 |
| MAP,  mmHg | 89.33  (83.00,97.58) | 94.33  (87.42,102.67) | 102.33  (92.00,111.67) | 111.33  （100,123.33） | <0.001 |
| FBG,mmol/L | 4.8(4.4,5.3) | 5.1(4.8,5.7) | 5.69(4.95,6.62) | 5.64（4.90,6.81） | <0.001 |
| FIN, uU/mL | 7.6(6.1,13.9) | 16.8(12.0,19.5) | 22.0(17.0,28.9) | 30（20.1,36.7） | <0.001 |
| HOMA-IR | 1.75(1.34,2.87) | 3.76(2.68,4.80) | 5.41(3.88,7.74) | 7.4（5.2,9.9） | <0.001 |
| TC, mmol/L | 3.96(3.53,4.53) | 4.53(4.08,5.12) | 5.20（4.54,5.72） | 5.38（4.89,6.14） | <0.001 |
| TG,mmol/L | 1.13(0.76,1.54) | 1.42(0.96,1.96) | 1.75（1.32,2.58） | 2.00（1.42,2.87） | <0.001 |
| HDL,mmol/L | 1.1(0.93,1.29) | 1.1(0.96,1.26) | 1.11（0.96,1.29） | 1.11（0.98,1.25） | 0.428 |
| LDL,mmol/L | 2.35(1.90,2.85) | 2.7(2.23,3.20) | 3.10（2.57,3.68） | 3.29（2.91,3.89） | <0.001 |
| Non-HDL-C, mmol/L | 2.84(2.45,3.48) | 3.40(2.93,4.00) | 4.05（3.27,4.57） | 4.26（3.64,4.95） | <0.001 |
| TC/HDL-C | 3.64(3.05,4.49) | 4.09(3.38,4.92) | 4.64（3.94,5.38） | 4.89（4.07,5.72） | <0.001 |
| TG/HDL-C | 1.04(0.63,1.55) | 1.23(0.83,1.93) | 1.56（1.03,2.48） | 1.86（1.19,2.65） | <0.001 |
| LDL/HDL-C | 2.16(1.64,2.82) | 2.53(1.83,3.05) | 2.82（2.24,3.31） | 3.05（2.39,3.74） | <0.001 |
| AI | 2.64(2.05,3.49) | 3.09(2.38,3.92) | 3.64（2.94,4.38） | 3.89（3.07，4.72） | <0.001 |
| LCI | 9.92(5.47,16.25) | 17.38(7.89,28.50) | 25.48（15.79,40.43） | 33.93（19.31,54.49） | <0.001 |
| VAI | 1.55(0.95,2.69) | 2.13(1.28,3.30) | 2.50（1.65,3.74） | 2.72（1.85,4.12） | 0.002 |
| LAP | 33.85(17.90,53.34) | 48.66(29.32,85.46) | 74.0（46.2,119.2） | 94.11（61.25,155.29） | <0.001 |
| Hyperglycemia，% | 4.8 | 16.8 | 35 | 37.5 | <0.001 |
| Hyperinsulinemia, % | 27.4 | 70.7 | 90.7 | 93.5 | <0.001 |
| HOMA-IR≥2.5, % | 30.1 | 79.9 | 95.6 | 95.7 | <0.001 |
| Hyper TC % | 10.2 | 21.7 | 51.9 | 59.8 | <0.001 |
| Hyper TG,% | 16.1 | 35.9 | 52.5 | 64.7 | <0.001 |
| Hypo  HDL-C% | 55.4 | 61.4 | 65 | 66.3 | 0.083 |
| Hyper LDL-C % | 6.50 | 17.9 | 37.2 | 45.1 | <0.001 |
| CVD% | 4.8 | 8.7 | 7.1 | 11.4 | 0.052 |
| HBP% | 16.1 | 23.9 | 43.2 | 56.5 | <0.001 |

| **Correlation** | | | | | | | | | | |
| --- | --- | --- | --- | --- | --- | --- | --- | --- | --- | --- |
|  | | | Sex=male | age | NHR | WHR | BMI | STOPBang | MAP | pRED_3p |
| Spearman correlation Rho | Sex=male | Spearman Correlation | 1.000 | -.036 | .109^**^ | -.165^**^ | -.114^**^ | .414^**^ | .123^**^ | .193^**^ |
|  |  | Sig.(2-tailed) | . | .332 | .003 | .000 | .002 | .000 | .001 | .000 |
|  |  | N | 737 | 737 | 737 | 737 | 737 | 737 | 737 | 737 |
|  | age | Spearman Correlation | -.036 | 1.000 | .107^**^ | .136^**^ | -.125^**^ | .350^**^ | .201^**^ | .289^**^ |
|  |  | Sig.(2-tailed) | .332 | . | .003 | .000 | .001 | .000 | .000 | .000 |
|  |  | N | 737 | 737 | 737 | 737 | 737 | 737 | 737 | 737 |
|  | NHR | Spearman Correlation | .109^**^ | .107^**^ | 1.000 | .717^**^ | .649^**^ | .548^**^ | .373^**^ | .427^**^ |
|  |  | Sig.(2-tailed) | .003 | .003 | . | .000 | .000 | .000 | .000 | .000 |
|  |  | N | 737 | 737 | 737 | 737 | 737 | 737 | 737 | 737 |
|  | WHR | Spearman Correlation | -.165^**^ | .136^**^ | .717^**^ | 1.000 | .813^**^ | .399^**^ | .380^**^ | .437^**^ |
|  |  | Sig.(2-tailed) | .000 | .000 | .000 | . | .000 | .000 | .000 | .000 |
|  |  | N | 737 | 737 | 737 | 737 | 737 | 737 | 737 | 737 |
|  | BMI | Spearman Correlation | -.114^**^ | -.125^**^ | .649^**^ | .813^**^ | 1.000 | .371^**^ | .391^**^ | .453^**^ |
|  |  | Sig.(2-tailed) | .002 | .001 | .000 | .000 | . | .000 | .000 | .000 |
|  |  | N | 737 | 737 | 737 | 737 | 737 | 737 | 737 | 737 |
|  | STOPBang | Spearman Correlation | .414^**^ | .350^**^ | .548^**^ | .399^**^ | .371^**^ | 1.000 | .456^**^ | .555^**^ |
|  |  | Sig.(2-tailed) | .000 | .000 | .000 | .000 | .000 | . | .000 | .000 |
|  |  | N | 737 | 737 | 737 | 737 | 737 | 737 | 737 | 737 |
|  | MAP | Spearman Correlation | .123^**^ | .201^**^ | .373^**^ | .380^**^ | .391^**^ | .456^**^ | 1.000 | .549^**^ |
|  |  | Sig.(2-tailed) | .001 | .000 | .000 | .000 | .000 | .000 | . | .000 |
|  |  | N | 737 | 737 | 737 | 737 | 737 | 737 | 737 | 737 |
|  | pRED_3p | Spearman Correlation | .193^**^ | .289^**^ | .427^**^ | .437^**^ | .453^**^ | .555^**^ | .549^**^ | 1.000 |
|  |  | Sig.(2-tailed) | .000 | .000 | .000 | .000 | .000 | .000 | .000 | . |
|  |  | N | 737 | 737 | 737 | 737 | 737 | 737 | 737 | 737 |
| **. Correlation is significant at the 0.01 level (2-tailed). | | | | | | | | | | |

**Table S11** The result of Spearman correlation(pRED_3p)

**Table S12** The result of Spearman correlation(SBII)

| **Correlation** | | | | | | | | | | |
| --- | --- | --- | --- | --- | --- | --- | --- | --- | --- | --- |
|  | | | Sex=男 | age | NHR | WHR | BMI | STOPBang | MAP | SBII |
| Spearman correlation Rho | Sex=male | Spearman Correlation | 1.000 | -.036 | .109^**^ | -.165^**^ | -.114^**^ | .414^**^ | .123^**^ | .196^**^ |
|  |  | Sig.(2-tailed) | . | .332 | .003 | .000 | .002 | .000 | .001 | .000 |
|  |  | N | 737 | 737 | 737 | 737 | 737 | 737 | 737 | 737 |
|  | age | Spearman Correlation | -.036 | 1.000 | .107^**^ | .136^**^ | -.125^**^ | .350^**^ | .201^**^ | .277^**^ |
|  |  | Sig.(2-tailed) | .332 | . | .003 | .000 | .001 | .000 | .000 | .000 |
|  |  | N | 737 | 737 | 737 | 737 | 737 | 737 | 737 | 737 |
|  | NHR | Spearman Correlation | .109^**^ | .107^**^ | 1.000 | .717^**^ | .649^**^ | .548^**^ | .373^**^ | .434^**^ |
|  |  | Sig.(2-tailed) | .003 | .003 | . | .000 | .000 | .000 | .000 | .000 |
|  |  | N | 737 | 737 | 737 | 737 | 737 | 737 | 737 | 737 |
|  | WHR | Spearman Correlation | -.165^**^ | .136^**^ | .717^**^ | 1.000 | .813^**^ | .399^**^ | .380^**^ | .443^**^ |
|  |  | Sig.(2-tailed) | .000 | .000 | .000 | . | .000 | .000 | .000 | .000 |
|  |  | N | 737 | 737 | 737 | 737 | 737 | 737 | 737 | 737 |
|  | BMI | Spearman Correlation | -.114^**^ | -.125^**^ | .649^**^ | .813^**^ | 1.000 | .371^**^ | .391^**^ | .461^**^ |
|  |  | Sig.(2-tailed) | .002 | .001 | .000 | .000 | . | .000 | .000 | .000 |
|  |  | N | 737 | 737 | 737 | 737 | 737 | 737 | 737 | 737 |
|  | STOPBang | Spearman Correlation | .414^**^ | .350^**^ | .548^**^ | .399^**^ | .371^**^ | 1.000 | .456^**^ | .558^**^ |
|  |  | Sig.(2-tailed) | .000 | .000 | .000 | .000 | .000 | . | .000 | .000 |
|  |  | N | 737 | 737 | 737 | 737 | 737 | 737 | 737 | 737 |
|  | MAP | Spearman Correlation | .123^**^ | .201^**^ | .373^**^ | .380^**^ | .391^**^ | .456^**^ | 1.000 | .555^**^ |
|  |  | Sig.(2-tailed) | .001 | .000 | .000 | .000 | .000 | .000 | . | .000 |
|  |  | N | 737 | 737 | 737 | 737 | 737 | 737 | 737 | 737 |
|  | SBII | Spearman Correlation | .196^**^ | .277^**^ | .434^**^ | .443^**^ | .461^**^ | .558^**^ | .555^**^ | 1.000 |
|  |  | Sig.(2-tailed) | .000 | .000 | .000 | .000 | .000 | .000 | .000 | . |
|  |  | N | 737 | 737 | 737 | 737 | 737 | 737 | 737 | 737 |
| **. . Correlation is significant at the 0.01 level (2-tailed).。 | | | | | | | | | | |

**Table S13** The result of Spearman correlation(AHI)

| **Correlation** | | | | | | | | | | |
| --- | --- | --- | --- | --- | --- | --- | --- | --- | --- | --- |
|  | | | Sex=male | age | NHR | WHR | BMI | STOPBang | MAP | AHI |
| Spearman correlation Rho | Sex=male | Spearman Correlation | 1.000 | -.036 | .109^**^ | -.165^**^ | -.114^**^ | .414^**^ | .123^**^ | .205^**^ |
|  |  | Sig.(2-tailed) | . | .332 | .003 | .000 | .002 | .000 | .001 | .000 |
|  |  | N | 737 | 737 | 737 | 737 | 737 | 737 | 737 | 737 |
|  | age | Spearman Correlation | -.036 | 1.000 | .107^**^ | .136^**^ | -.125^**^ | .350^**^ | .201^**^ | .268^**^ |
|  |  | Sig.(2-tailed) | .332 | . | .003 | .000 | .001 | .000 | .000 | .000 |
|  |  | N | 737 | 737 | 737 | 737 | 737 | 737 | 737 | 737 |
|  | NHR | Spearman Correlation | .109^**^ | .107^**^ | 1.000 | .717^**^ | .649^**^ | .548^**^ | .373^**^ | .411^**^ |
|  |  | Sig.(2-tailed) | .003 | .003 | . | .000 | .000 | .000 | .000 | .000 |
|  |  | N | 737 | 737 | 737 | 737 | 737 | 737 | 737 | 737 |
|  | WHR | Spearman Correlation | -.165^**^ | .136^**^ | .717^**^ | 1.000 | .813^**^ | .399^**^ | .380^**^ | .399^**^ |
|  |  | Sig.(2-tailed) | .000 | .000 | .000 | . | .000 | .000 | .000 | .000 |
|  |  | N | 737 | 737 | 737 | 737 | 737 | 737 | 737 | 737 |
|  | BMI | Spearman Correlation | -.114^**^ | -.125^**^ | .649^**^ | .813^**^ | 1.000 | .371^**^ | .391^**^ | .406^**^ |
|  |  | Sig.(2-tailed) | .002 | .001 | .000 | .000 | . | .000 | .000 | .000 |
|  |  | N | 737 | 737 | 737 | 737 | 737 | 737 | 737 | 737 |
|  | STOPBang | Spearman Correlation | .414^**^ | .350^**^ | .548^**^ | .399^**^ | .371^**^ | 1.000 | .456^**^ | .525^**^ |
|  |  | Sig.(2-tailed) | .000 | .000 | .000 | .000 | .000 | . | .000 | .000 |
|  |  | N | 737 | 737 | 737 | 737 | 737 | 737 | 737 | 737 |
|  | MAP | Spearman Correlation | .123^**^ | .201^**^ | .373^**^ | .380^**^ | .391^**^ | .456^**^ | 1.000 | .536^**^ |
|  |  | Sig.(2-tailed) | .001 | .000 | .000 | .000 | .000 | .000 | . | .000 |
|  |  | N | 737 | 737 | 737 | 737 | 737 | 737 | 737 | 737 |
|  | AHI | Spearman Correlation | .205^**^ | .268^**^ | .411^**^ | .399^**^ | .406^**^ | .525^**^ | .536^**^ | 1.000 |
|  |  | Sig.(2-tailed) | .000 | .000 | .000 | .000 | .000 | .000 | .000 | . |
|  |  | N | 737 | 737 | 737 | 737 | 737 | 737 | 737 | 737 |
| **. . Correlation is significant at the 0.01 level (2-tailed).。 | | | | | | | | | | |

**Table S14** The result of Collinearity Diagnostics (pRED_3p)

| **Coefficient^a^** | | | | | | | | | | |
| --- | --- | --- | --- | --- | --- | --- | --- | --- | --- | --- |
| Model | | Unstandardized Coefficients | | Standardized Coefficients | t | Sig. | The 95% CI Oof B | | Collinearity Statistics | |
|  |  | B | Std.Error | Beta |  |  | lower limits | Upper  limits | Tolerance | VIF |
| 1 | (Constant) | 2.823 | .548 |  | 5.153 | .000 | 1.748 | 3.899 |  |  |
|  | Sex=male | -.010 | .123 | -.003 | -.077 | .938 | -.251 | .232 | .614 | 1.629 |
|  | age | .012 | .004 | .139 | 3.236 | .001 | .005 | .019 | .636 | 1.571 |
|  | BMI | .020 | .011 | .114 | 1.909 | .057 | -.001 | .041 | .333 | 3.007 |
|  | NHR | 1.569 | 2.491 | .031 | .630 | .529 | -3.323 | 6.460 | .482 | 2.076 |
|  | WHR | 1.142 | .747 | .090 | 1.529 | .127 | -.324 | 2.609 | .344 | 2.906 |
|  | STOPBang | .050 | .041 | .065 | 1.218 | .224 | -.031 | .131 | .418 | 2.394 |
|  | MAP | .003 | .004 | .035 | .820 | .412 | -.004 | .010 | .631 | 1.584 |
|  | pRED_3p | .808 | .290 | .122 | 2.786 | .005 | .239 | 1.377 | .612 | 1.634 |
| a. Dependent Variable: FBG | | | | | | | | | | |

**Table S15** The result of Collinearity Diagnostics (pRED_3p)

| Collinearity Diagnostics.**^a^** | | | | | | | | | | | | |
| --- | --- | --- | --- | --- | --- | --- | --- | --- | --- | --- | --- | --- |
| Model | Dimension | Eigenvalue | Condition Index | Variance Proportions | | | | | | | | |
|  |  |  |  | (Constant) | Sex=  mae | age | BMI | NHR | WHR | STOPBang | MAP | pRED_3p |
| 1 | 1 | 7.902 | 1.000 | .00 | .00 | .00 | .00 | .00 | .00 | .00 | .00 | .00 |
|  | 2 | .513 | 3.924 | .00 | .00 | .00 | .00 | .00 | .00 | .00 | .00 | .57 |
|  | 3 | .318 | 4.984 | .00 | .53 | .01 | .00 | .00 | .00 | .01 | .00 | .07 |
|  | 4 | .127 | 7.876 | .00 | .02 | .33 | .02 | .00 | .00 | .13 | .00 | .04 |
|  | 5 | .096 | 9.055 | .00 | .09 | .14 | .02 | .00 | .00 | .55 | .00 | .12 |
|  | 6 | .021 | 19.595 | .03 | .22 | .29 | .23 | .00 | .05 | .11 | .33 | .01 |
|  | 7 | .011 | 26.235 | .04 | .10 | .19 | .47 | .13 | .11 | .06 | .33 | .12 |
|  | 8 | .008 | 32.206 | .22 | .03 | .01 | .25 | .03 | .65 | .01 | .25 | .04 |
|  | 9 | .004 | 46.469 | .71 | .00 | .04 | .01 | .83 | .20 | .13 | .09 | .03 |
| a. Dependent Variable: FBG | | | | | | | | | | | | |

**Table S16** The result of Collinearity Diagnostics (SBII)

| **Coefficient^a^** | | | | | | | | | | |
| --- | --- | --- | --- | --- | --- | --- | --- | --- | --- | --- |
| Model | | Unstandardized Coefficients | | Standardized Coefficients | t | Sig. | The 95% CI Oof B | | Collinearity Statistics | |
|  |  | B | Std.Error | Beta |  |  | lower limits | Upper  limits | Tolerance | VIF |
| 1 | (Constant) | 2.624 | .540 |  | 4.858 | .000 | 1.564 | 3.684 |  |  |
|  | Sex=男 | .006 | .123 | .002 | .052 | .959 | -.235 | .248 | .616 | 1.623 |
|  | age | .013 | .004 | .153 | 3.560 | .000 | .006 | .020 | .646 | 1.548 |
|  | BMI | .022 | .011 | .125 | 2.101 | .036 | .001 | .043 | .336 | 2.980 |
|  | NHR | 1.627 | 2.499 | .032 | .651 | .515 | -3.279 | 6.533 | .481 | 2.077 |
|  | WHR | 1.075 | .749 | .084 | 1.436 | .151 | -.394 | 2.545 | .344 | 2.904 |
|  | STOPBang评分 | .059 | .041 | .077 | 1.447 | .148 | -.021 | .140 | .423 | 2.363 |
|  | MAP | .005 | .004 | .054 | 1.275 | .203 | -.002 | .012 | .660 | 1.515 |
|  | SBII | .001 | .001 | .079 | 1.970 | .049 | .000 | .002 | .729 | 1.371 |
| a. Dependent Variable: FBG | | | | | | | | | | |

**Table S17** The result of Collinearity Diagnostics (SBII)

| Collinearity Diagnostics**^a^** | | | | | | | | | | | | |
| --- | --- | --- | --- | --- | --- | --- | --- | --- | --- | --- | --- | --- |
| Model | Dimension | Eigenvalue | Condition Index | Variance Proportions | | | | | | | | |
|  |  |  |  | (Constant) | Sex=  male | age | BMI | NHR | WHR | STOPBang | MAP | SBII |
| 1 | 1 | 7.687 | 1.000 | .00 | .00 | .00 | .00 | .00 | .00 | .00 | .00 | .00 |
|  | 2 | .715 | 3.280 | .00 | .00 | .00 | .00 | .00 | .00 | .00 | .00 | .72 |
|  | 3 | .323 | 4.881 | .00 | .52 | .01 | .00 | .00 | .00 | .01 | .00 | .04 |
|  | 4 | .133 | 7.609 | .00 | .05 | .25 | .02 | .00 | .00 | .21 | .00 | .01 |
|  | 5 | .099 | 8.809 | .00 | .08 | .23 | .02 | .00 | .00 | .43 | .00 | .08 |
|  | 6 | .021 | 19.325 | .03 | .22 | .29 | .23 | .00 | .05 | .10 | .35 | .01 |
|  | 7 | .012 | 25.275 | .05 | .10 | .17 | .44 | .12 | .09 | .09 | .34 | .09 |
|  | 8 | .008 | 31.679 | .22 | .02 | .01 | .29 | .02 | .66 | .01 | .23 | .03 |
|  | 9 | .004 | 45.539 | .69 | .00 | .03 | .01 | .85 | .20 | .15 | .08 | .01 |
| a. Dependent Variable: FBG | | | | | | | | | | | | |

**Table S18** The result of Collinearity Diagnostics (AHI)

| **Coefficient^a^** | | | | | | | | | | |
| --- | --- | --- | --- | --- | --- | --- | --- | --- | --- | --- |
| Model | | Unstandardized Coefficients | | Standardized Coefficients | t | Sig. | The 95% CI Oof B | | Collinearity Statistics | |
|  |  | B | Std.Error | Beta |  |  | lower limits | Upper  limits | Tolerance | VIF |
| 1 | (Constant) | 3.048 | .549 |  | 5.551 | .000 | 1.970 | 4.125 |  |  |
|  | Sex=男 | -.029 | .122 | -.010 | -.238 | .812 | -.270 | .211 | .613 | 1.632 |
|  | age | .011 | .004 | .133 | 3.108 | .002 | .004 | .018 | .636 | 1.572 |
|  | BMI | .015 | .011 | .084 | 1.387 | .166 | -.006 | .036 | .322 | 3.109 |
|  | NHR | 1.486 | 2.479 | .030 | .600 | .549 | -3.380 | 6.353 | .482 | 2.075 |
|  | WHR | 1.167 | .743 | .091 | 1.570 | .117 | -.292 | 2.626 | .344 | 2.906 |
|  | STOPBang评分 | .047 | .041 | .061 | 1.157 | .248 | -.033 | .128 | .421 | 2.377 |
|  | MAP | .002 | .004 | .021 | .489 | .625 | -.005 | .009 | .640 | 1.562 |
|  | AHI | .008 | .002 | .174 | 3.885 | .000 | .004 | .013 | .582 | 1.717 |
| a. Dependent Variable: FBG | | | | | | | | | | |

**Table S19** The result of Collinearity Diagnostics (AHI)

| Collinearity Diagnostics**^a^** | | | | | | | | | | | | |
| --- | --- | --- | --- | --- | --- | --- | --- | --- | --- | --- | --- | --- |
| Model | Dimension | Eigenvalue | Condition Index | Variance Proportions | | | | | | | | |
|  |  |  |  | (Constant) | Sex=  male | age | BMI | NHR | WHR | STOPBang | MAP | AHI |
| 1 | 1 | 8.012 | 1.000 | .00 | .00 | .00 | .00 | .00 | .00 | .00 | .00 | .00 |
|  | 2 | .399 | 4.482 | .00 | .01 | .01 | .00 | .00 | .00 | .01 | .00 | .51 |
|  | 3 | .323 | 4.980 | .00 | .51 | .00 | .00 | .00 | .00 | .01 | .00 | .09 |
|  | 4 | .131 | 7.830 | .00 | .04 | .30 | .02 | .00 | .00 | .15 | .00 | .02 |
|  | 5 | .092 | 9.350 | .00 | .09 | .18 | .02 | .00 | .00 | .54 | .00 | .16 |
|  | 6 | .021 | 19.665 | .03 | .21 | .27 | .20 | .00 | .05 | .09 | .36 | .00 |
|  | 7 | .011 | 26.724 | .04 | .11 | .20 | .52 | .13 | .10 | .06 | .30 | .14 |
|  | 8 | .008 | 32.349 | .21 | .03 | .01 | .22 | .04 | .65 | .01 | .24 | .04 |
|  | 9 | .004 | 46.941 | .71 | .00 | .04 | .01 | .83 | .20 | .14 | .10 | .03 |
| a. Dependent Variable: FBG | | | | | | | | | | | | |

**Table S20** The result of Spearman correlation(pRED_3p and SBII)

| **Correlation** | | | | |
| --- | --- | --- | --- | --- |
|  | | | pRED_3p | SBII |
| Spearman  Correlation  Rho | pRED_3p | Spearman  Correlation | 1.000 | .992^**^ |
|  |  | Sig.（2-tailed） | . | .000 |
|  |  | N | 737 | 737 |
|  | SBII | Spearman  Correlation | .992^**^ | 1.000 |
|  |  | Sig.（2-tailed） | .000 | . |
|  |  | N | 737 | 737 |
| **. . Correlation is significant at the 0.01 level (2-tailed).。 | | | | |

**Table S21** The resuilt of the Kolmogorov-Smirnov test

| Variable | Number of cases | K-S Test Statistic | Significance(2-tailed) |
| --- | --- | --- | --- |
| age | 737 | 0.095 | .000c |
| NHR | 737 | 0.118 | .000c |
| WHR | 737 | 0.095 | .000c |
| BMI | 737 | 0.117 | .000c |
| STOP-Bang | 737 | 0.12 | .000c |
| pRED_3p | 737 | 0.201 | .000c |
| SBII | 737 | 0.294 | .000c |
| AHI | 737 | 0.146 | .000c |
| MAP | 737 | 0.064 | .000c |
| FBG(mmol/L) | 737 | 0.135 | .000c |
| FIN（mU/L） | 737 | 0.153 | .000c |
| HOMA-IR | 737 | 0.204 | .000c |
| TC | 737 | 0.044 | .002c |
| TG | 737 | 0.167 | .000c |
| HDL-C | 737 | 0.07 | .000c |
| LDL-C | 737 | 0.05 | .000c |
| Non HDL-C | 737 | 0.05 | .000c |
| TC/HDL-C | 737 | 0.13 | .000c |
| TG/HDL-C | 737 | 0.303 | .000c |
| LDL-C/HDL-C | 737 | 0.05 | .000c |
| AI | 737 | 0.13 | .000c |
| LCI | 737 | 0.353 | .000c |
| VAI | 737 | 0.297 | .000c |
| LAP | 737 | 0.151 | .000c |
